# A neglected case of giant synovial chondromatosis in knee joint

**DOI:** 10.11604/pamj.2015.22.5.7481

**Published:** 2015-09-03

**Authors:** Sancar Serbest, Ugur Tiftikçi, Fatih Karaaslan, Haci Bayram Tosun, Hüseyin Fatih Sevinç, Mahi Balci

**Affiliations:** 1 Department of Orthopaedics and Traumatology, Faculty of Medicine, Kirikkale University, Kirikkale, Turkey; 2 Department of Orthopaedics and Traumatology, Faculty of Medicine, Bozok University, Yozgat, Turkey; 3 Department of Orthopaedics and Traumatology, Faculty of Medicine, Adiyaman University, Adiyaman, Turkey; 4 Department of Pathology, Faculty of Medicine, Kirikkale University, Kirikkale, Turkey

**Keywords:** Synovial chondromatosis, giant, loose body, knee Joint, surgery

## Abstract

Synovial chondromatosis is a rare benign condition arising from the synovial membrane of the joints, synovial sheaths or bursae around the joints. Primary synovial chondromatosis typically affects the large joints in the third to fifth decade of life. The purpose of this case report is to document this rare synovial pathology, which required open synovectomy and debridement to eradicate it. In our case, the biggest sized SOC was 20x19x6 cm, although there were many joint mice. Our case had the biggest SOC ever extracted, which to the best of my knowledge has not been reported earlier.

## Introduction

Synovial osteochondromatosis (SOC) is a monoarticular, synovial, proliferative disease. It is a rare disease which presents as multiple cartilaginous nodules in synovial joints, bursae or tendon sheaths [1–3]. Although it is generally a primary condition, it can be secondary to osteoarthritis. This disease is more prevalent in 4^th^ and 5^th^ decades. It affects males more than females [1]. SOC most commonly involves knee joint with a frequency of 50-65%. Other places that are involved frequently include hip, elbow, shoulder, and ankle. Monoarticular involvement occurs. Observed clinical symptoms were pain (85-100% of cases), swelling (42-58% of cases) and limitation of joint movements (38-55%) at the involved area. Secondary osteoarthritis findings such as generalized joint effusions, locking, tenderness, and crepitation may also occur [2, 4]. Therefore early diagnosis and treatment of this disease is important. Although rare, SOC should be considered in differential diagnosis of cases including joint pathology. To the best of our knowledge a SOC case at this amount has never been reported. In this article we present a case of intraarticular SOC at knee joint that was successfully treated with knee arthrotomy.

## Patient and observation

**Figure 1:**
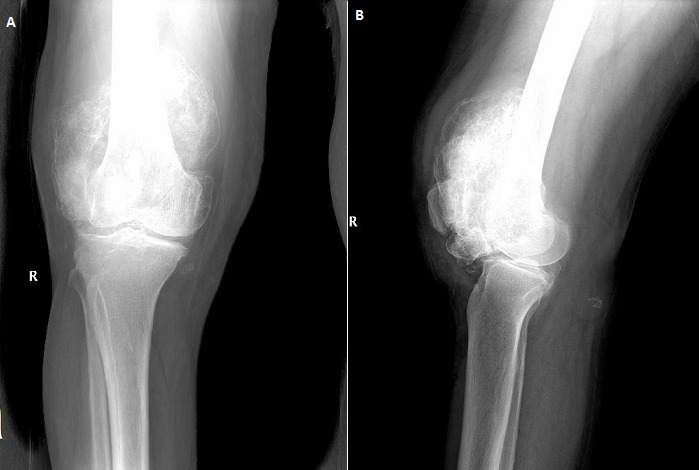
X-ray of right knee joint showing the radiodence body. Anteroposterior (a) and lateral (b) X-ray of the knee

**Figure 2:**
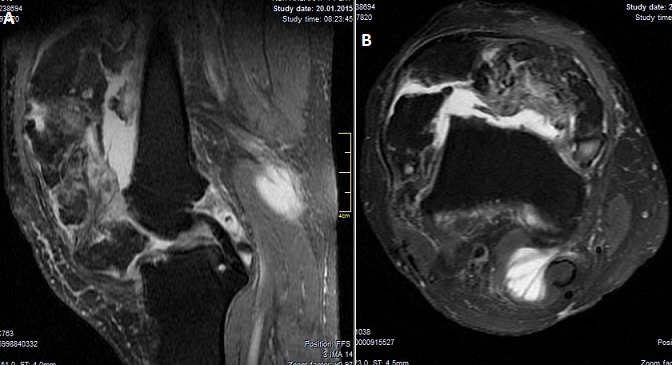
MRI T-2 image showing the effusion, synovial hypertrophy and a loose calcific body (a, b)

A seventy two years old male presented to our clinic with swelling, pain, and limitation of movement at knee joint. The patient's history revealed swellings and pain at knee joint for nearly last 20 years. He was prescribed nonsteroidal anti-inflammatory drugs and rest. But pain didn't relieve and limitations in range of motion occurred at knee joint. Physical examination findings included a solid and semi-mobile mass at anterior knee region. Knee joint range of motion was 80 degrees flexion and 0 degrees extension. There wasn't any neurovascular pathology. Antero-posterior and lateral knee X-rays showed degenerative changes and a giant, irregular, calcific mass which started below patella and extended to suprapatellar area (Figure 1). Knee MRI revealed many loose bodies (consistent with synovial chondromatosis) isointense with fatty bone marrow at all sequences at the level of right knee joint. Scalloping was observed at tibial and femoral surfaces (Figure 2).

**Figure 3:**
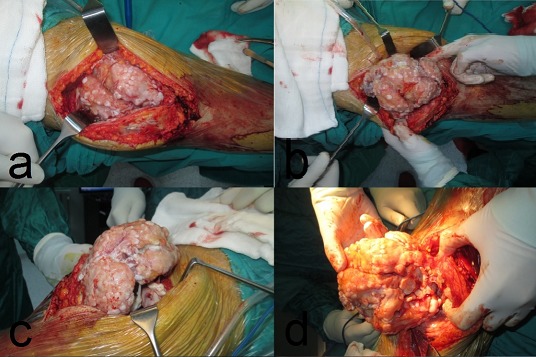
Right knee joint exposed by medial parapatellar approach, a large loose body lying in front of intercondylar notch (a, b, c, d)

**Figure 4:**
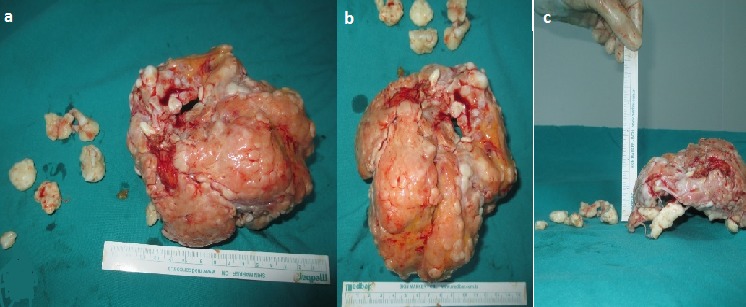
A large loose body of around 20x19x6 cm extracted from the knee joint (a, b c)

**Figure 5:**
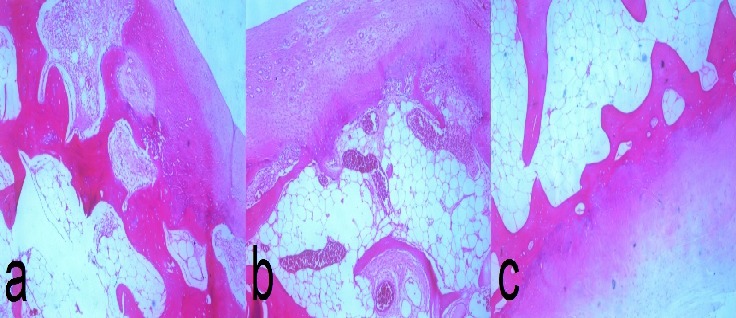
Histopathology slide of the loose body showing lobules of cartilage without cellular atypia (a, b c)

**Figure 6:**
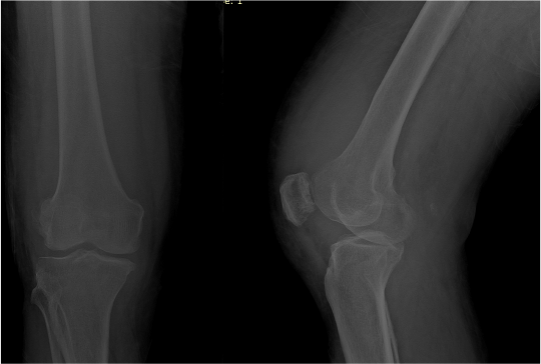
Radiographic findings after the removal of large loose body. Anteroposterior (a) and lateral (b) X-ray of the knee

Decision for a surgical intervention was made and after informing the patient, consent was taken. Under spinal anesthesia, a tourniquet was applied to right lower extremity. Right knee was entered with an anterior longitudinal incision. Cutaneous and subcutaneous tissues were passed. Quadriceps mechanism was opened with a medial parapatellar incision and patella was toppled to left side. Intraarticular mass was exposed (Figure 3). The mass was released from surrounding tissues by blunt excision and excised with enucleation (Figure 4). Then synovial tissue was excised with synoviectomy. Surgical area was irrigated using abundant isotonic solution. The excised mass was sent to pathology laboratory. Pathology evaluation showed that the mass was consistent with synovial chondromatosis (Figure 5). Active and passive exercises were started at an early period after surgery. Recurrence was not detected at his last control visit. Knee movements were at full range when compared with the other knee. There was no pain or other complaints (Figure 6).

## Discussion

Synovial chondromatosis is a rare, monoarticular benign neoplasm with an unknown cause. It can be originated from any joint, tendon sheath, or bursae that have synovial tissue. It is characterized by cartilaginous nodule formation secondary to synovial metaplasia. These nodules may be calcified apart from synovial tissue. Although it is generally progressive in nature, it can limit itself and regress [3]. Transformation to malignancy is reported to be very low (5%). Delay in diagnosis of synovial chondromatosis pathology may occur due to slow progression of disease and calcification of free cartilage fragments at later stages [5]. Complaints of the patients are usually due to mechanical effects of free bodies. Milgram [2], defined three stages of this disease: Stage 1 (early stage) is the active intrasynovial stage during which there is no free articular body. Stage II is the transition stage from intrasynovial disease to free bodies. In this stage there are both active intrasynovial disease and free bodies. In stage III (late stage) there are multiple free bodies in the absence of intrasynovial involvement. When our case was diagnosed the disease was at stage III.

Radiologic features of disease vary according to stages. At first stage there is swelling only around the involved joint [6]. The most common radiologic finding, radiopaque free bodies with varying sizes, can be seen at any place in the joint cavity [7]. Calcification occurs at last stage and may not be observed in every patient [8]. Intraarticular liquid like masses, non-calcified masses or swellings which can be septated may be differentiated with magnetic resonance imaging (MRI) or computed tomography (CT) [9]. Especially MRI gets multiplanar sequences and is a very important diagnostic tool to evaluate soft tissues. Matsumoto et al [10] reported that MRI is much more helpful than CT. But hardness of diagnosis despite MRI should also be considered. In neglected cases or in cases with a long term disease, changes in bone or joint cartilage induced by multiple intraarticular lesions, bony erosion, or presence of local osteoporosis make diagnosis more difficult [11].

Differential diagnosis of synovial chondromatosis includes many diseases. Therefore it should be differentiated from benign lesions such as synovial hemangioma, pigmented villonodular synovitis, synovial cyst, lipoma arborescence and from malign lesions such as synovial chondrosarcoma and synovial sarcoma [12]. Malignant transformation was reported in a few cases at long term follow up [13]. Anract et al [14] reported that malign transformation is very rare and this condition should be suspected when there is bone involvement in MRI and a rapid progression of clinical picture. Big and hard lesions are much rarer than multiple small lesions. If the lesion size is big and patient's age is at later decades differential diagnosis should be made with more prevalent chondrosarcomas [15, 16]. In our case it was easier with MRI to diagnose the intraarticular big mass which was seen with calcifications in X-ray films. However, differential diagnosis from conditions such as chondrosarcoma is very important. In literature early artroscopic or open debridement, synoviectomy, and removal of free cartilage pieces at an early stage before cartilage damage occurs have shown to be efficient treatments [15–17]. Severity of disease and affected location should be considered when deciding the treatment [15]. Additionally, in some cases that had osteochondroplasty after debridement osteoarthritis did not occur in two years follow up and successful results were achieved [7]. We preferred open surgery due to the size of the mass.

In our case, the biggest sized SOC was 20x19x6 cm, although there were many joint mice. In our literature search we found that the biggest masses that have been extracted till now were 10.5x5.5x5.5 cm [18], 6x4x4 cm [19], 5.3x3x2.3 cm [20], and our case had the biggest SOC ever extracted.

## Conclusion

In conclusion, SOC with monoarticular involvement is a very rare disease. Clinical diagnosis is very difficult. Consistency of clinical, radiological and histological findings should be sought in every case. After the diagnosis synovial excision as complete as possible, extraction of free bodies, and a close follow up are warranted.
